# Use of single‐use ultra‐slim endoscopes for evaluation of pediatric esophageal varices: A pilot feasibility study

**DOI:** 10.1002/jpr3.70220

**Published:** 2026-07-13

**Authors:** Vishruti Patel, Evan Greenhall, Eric Dybbro, Sheetal Wadera, Joel A. Friedlander, Shauna Schroeder, Paul Tran

**Affiliations:** ^1^ Division of Gastroenterology Phoenix Children's Hospital Phoenix Arizona USA; ^2^ Division of Gastroenterology, College of Medicine University of Arizona Phoenix Arizona USA

**Keywords:** portal hypertension, sedation‐free, transnasal endoscopy

## Abstract

Esophageal varices (EV) need monitoring in patients with portal hypertension. In 2022, a single‐use ultra‐slim (3.5 mm) gastroscope was approved for use in children and adults. This study evaluated if an ultra‐slim gastroscope could evaluate, grade, and corroborate the presence of EV in sedated pediatric patients as compared to standard oral gastroscopes (>8.6 mm). Ten pediatric subjects with suspected or known EV underwent surveillance endoscopy using the ultra‐slim endoscope followed by the standard gastroscope under sedation. Esophageal findings were analyzed using images from both procedures by two independent endoscopists. Visual findings for the presence or absence of EV had 100% congruence. EV were identified in 8/10 subjects (Grade I (4), Grade II (3), and Grade II/III (1)). Endoscopic grading matched between both endoscopes in 7/8 (87.5%) cases. Ultra‐slim and standard gastroscopes showed reliable visual agreement and could be used similarly in certain clinical settings to evaluate EV.

## Abstract (highlights)

**What is Known:**

- Endoscopy with a larger standard oral gastrosocope has been used as a gold standard for diagnosis, monitoring, and management of esophageal varices (EV).
- Standard endoscopy requires sedation, which increases anesthesia exposure in patients who are solely being screened for complications of portal hypertension.
- Sedation‐free transnasal endoscopy (TNE) has been used in the diagnostic evaluation of multiple diseases (eosinophilic esophagitis, dysphagia, etc.).

**What is New:**

- Transoral and/or TNE with a newly cleared Food and Drug Administration (FDA) pediatric single‐use ultra‐slim endoscope can accurately and reliably assess the presence of and grade EV and corroborates findings using larger diameter endoscopes.

## 1 INTRODUCTION

In pediatric patients with portal hypertension, esophageal varices (EV) pose a potentially life‐threatening complication that requires frequent monitoring.^1^ Management of EV focuses on preventing a first‐time occurrence of variceal bleeding (primary prophylaxis) or preventing recurrent variceal bleeding (secondary prophylaxis).^2^ For secondary prophylaxis, pediatric patients often require surveillance esophagogastroduodenoscopy (EGD) every 2–4 weeks after a bleeding episode until eradication of EV, then routine surveillance EGD every 1–3 years.^2^, ^3^ Conversely, there is a lack of consensus on surveillance guidelines for primary prophylaxis of EV in pediatric patients.^3^ In general, most pediatric guidelines discourage primary prophylaxis screening unless a patient falls into a high‐risk subgroup, often based on clinical evidence of portal hypertension such as splenomegaly, thrombocytopenia, or degree of liver disease and dysfunction.^2^, ^3^

Endoscopy remains the gold standard for diagnosing and grading EV; however, use of sedation and cost associated with performing an oral EGD are increased risks for patients requiring frequent monitoring.^1^, ^4^ Sedation‐free capsule endoscopy and transnasal endoscopy (TNE) are some well‐studied, cost‐effective alternatives in adult medicine literature for the monitoring of EV.^5^, ^6^

Recently, in pediatrics, sedation‐free TNE was associated with reduced anesthesia risk, fewer adverse events, increased patient/family satisfaction, shorter procedure and recovery time, and increased access to endoscopy procedures.^4^, ^7^, ^8^, ^9^ A study by Lee et al. found sedation‐free TNE to be more cost‐effective than EGD, especially by avoiding sedation‐associated charges.^9^ Sedation‐free TNE is now recognized across the United States as an alternative to traditional endoscopy and has been successfully used in the diagnostic evaluation of eosinophilic esophagitis, gastroesophageal reflux disease, esophagitis, fungal esophagitis, dysphagia, celiac disease, and Barrett's esophagus.^10^, ^11^, ^12^ To date, there have been no pediatric reports in the literature outside societal abstracts for its use in monitoring EV, but such use has been noted in adult gastroenterology.^1^, ^8^, ^11^, ^12^ The adult manuscripts report the use of ultrathin endoscopic devices with outer diameters exceeding 4.9 mm, which are thought to be unsuitable for pediatric nasal anatomy.^1^, ^11^, ^12^

In 2022, EvoEndo, Inc. received specific Food and Drug Administration (FDA) clearance for a single‐use ultra‐slim (3.5 mm outer diameter/2.0 mm working channel) gastroscope system specifically for transnasal or transoral endoscopy in patients greater than 5 years of age. In 2025, the FDA approved its use for all pediatric and adult age groups. While this device has been successfully used for pediatric diagnostic TNE, it was unclear if an ultra‐slim endoscope could deliver adequate quality to allow for precise grading of EV as compared to a much larger standard of care oral gastroscopes (>8.6 mm outer diameter).^13^, ^14^ This pilot study aimed to evaluate the use of this single‐use, ultra‐slim gastroscope for accurate and reliable identification of EV in pediatric patients and assess its corroboration with standard of care oral gastroscopes.

## 2 METHODS

### 2.1 Ethics statement

Phoenix Children's Hospital institutional review board (IRB‐23‐203) approval was obtained prior to study initiation. Informed consent and informed assent (where applicable) were obtained.

### 2.2 Study participants

Between December 2023 and September 2024, subjects under 18 years of age with a known history of EV scheduled for oral EGD for primary or secondary prophylaxis under anesthesia at Phoenix Children's Hospital were recruited. Subjects were hemodynamically stable and required no emergency interventions. Subjects with anatomy not compatible with TNE or with co‐diagnoses of eosinophilic esophagitis or erosive esophagitis were excluded. Subjects were voluntarily enrolled into the study via phone or clinic visit before their procedure.

### 2.3 Study procedure and data collection

With both the principal investigator (PI) and hepatologist present, subjects were sedated under general anesthesia. With the patient in supine position, the EvoEndo Model LE 110 cm single‐use gastroscope was introduced transnasally or transorally per patient preference by the PI. Any EVs identified were photographed and graded by the PI and hepatologist. The validated modified Paquet classification system was used to grade EVs as follows: Grade I varices are flattened by insufflation; Grade II varices are not flattened by insufflation and are separated by areas of normal mucosa; Grade III varices are not flattened by insufflation and are confluent or occupy more than 50% of the luminal circumference.^15^ The ultra‐slim gastroscope was then withdrawn, and a large diameter oral gastroscope (Olympus Q180, Olympus H190) was introduced by the hepatologist. Any varices identified were photographed and similarly graded by the PI and the hepatologist. Any subsequent therapeutic interventions such as variceal banding or sclerotherapy were performed with the standard oral gastroscope. Following the procedure, patients were transitioned to the post‐anesthesia care unit.

### 2.4 Outcome assessment

Images and videos of EV from the procedure were evaluated during and after the procedure by the PI and hepatologist for congruence of grading.

## 3 RESULTS

Between December 2023 and September 2024, 10 subjects were enrolled in this study. The median age was 16.2 years (range: 2.9–18.9 years). Although the EvoEndo scope was FDA‐approved for ages 5 and older at the time of the study, the smaller diameter EvoEndo scope was used off‐label in a 2.9‐year‐old child given concerns for difficult esophageal intubations on prior endoscopies. All subjects had liver disease and/or portal hypertension from various causes: congenital hepatic fibrosis (*n* = 1), biliary atresia post Kasai (*n* = 1), duplicated portal veins (*n* = 1), hepatoblastoma with cavernous transformation of portal vein (CTPV) (*n* = 1), metabolic dysfunction‐associated steatotic liver disease (MASLD) (*n* = 1), autoimmune hepatitis (AIH) with primary sclerosing cholangitis (PSC) (*n* = 1), AIH alone (*n* = 1), CTPV alone (*n* = 2), and Fontan‐associated liver disease (FALD) (*n* = 1). Endoscopy was performed for primary prophylaxis in five subjects and secondary prophylaxis in the remaining subjects. Endoscopic entry route was split evenly between transoral (*n* = 5) and transnasal (*n* = 5). Table 1 highlights subject and procedural characteristics.

**Table 1:** Patient and procedural characteristics.

Patient	Age (years)	Sex	Diagnosis	Indication for endoscopy	Last known esophageal varices	Route of TNE entry	Varices grade via ultra‐slim gastroscope	Varices grade via standard gastroscope	Intervention performed	Prior interventions
1	18.9	M	Congenital hepatic fibrosis	Primary prophylaxis	Grade I (14 months ago)	Nasal	Grade I	Grade I	None	Band ligation ×1 (last performed 8 years ago)
2	16.5	M	Biliary atresia	Primary prophylaxis	History of varices, record not available	Oral	Grade I	Grade I	None	Band ligation (×1) (no record of timing)
3	10.4	F	Duplicated portal vein	Primary prophylaxis	Grade I (9 months ago)	Oral	Grade I	Grade I	None	Band ligation ×3 (last performed 2 years ago) On NSBB
4	8.4	M	Hepatoblastoma with CTPV	Secondary prophylaxis (EV bleed about 3.5 months ago)	Grade I (3.5 months ago)	Oral	Grade I	Grade I	None	Sclerotherapy ×1 (last performed 2 years ago) Band ligation ×1 (last performed 1 year ago)
5	15.8	M	Metabolic dysfunction‐associated steatotic liver disease	Primary prophylaxis	Grade II (2 months ago)	Nasal	None (esophagitis)	None (esophagitis)	None	None
6	17.3	M	AIH and primary sclerosing cholangitis	Secondary prophylaxis (presenting with recurrent EV bleed symptoms)	Grade III ×2 Grade II ×1 (1 month ago)	Nasal	Grade III ×2 Grade II ×1	Grade III ×2 Grade II ×1	Band ligation	Band ligation ×2 (last performed 1 month ago)
7	17.2	M	AIH	Primary prophylaxis	Grade II ×3 Grade III ×1 (2 months ago)	Nasal	Grade II x3	Grade II ×3	Band ligation	Band ligation ×2 (last performed 2 months ago)
8	2.9	F	CTPV	Secondary prophylaxis (presenting with recurrent EV bleed symptoms)	Grade III ×4 (1 month ago)	Oral	Grade I	Grade II ×4	Sclerotherapy	Sclerotherapy ×1 (last performed 1 month ago) on NSBB
9	16.8	M	Fontan‐associated liver disease	Secondary prophylaxis (EV bleed 3 years ago)	Grade II (13 months ago)	Nasal	None (normal mucosa)	None (normal mucosa)	None	Band ligation (last performed 3 years ago)
10	8.3	M	CTPV	Secondary prophylaxis (EV bleed 2 years ago)	Grade II ×2 (6 months ago)	Oral	Grade II ×1	Grade II ×1	None	Band ligation ×2 (last performed 1 year ago) on NSBB

Abbreviations: AIH, autoimmune hepatitis; CTPV, cavernous transformation of portal vein; EV, esophageal varices; NSBB, non‐selective beta blocker; TNE, transnasal endoscopy.

EVs were identified in 8/10 subjects. Two subjects previously diagnosed with EV had no EV identified with both endoscopy methods. One was identified to have esophagitis, and the other had normal mucosa identified by both endoscopy methods. This reflects 100% visual congruence. Of the remaining eight subjects with EV, presence was confirmed on both ultra‐slim and standard gastroscope with 100% congruence. Grading of varices matched in 7/8 (87.5%) cases. One case had Grade I varices with the ultra‐slim gastroscope but multiple Grade II varices per the standard gastroscope. Otherwise, four subjects had Grade I varices, two subjects had Grade II varices, and one subject had a mix of Grade II and Grade III varices. Figure 1 shows esophageal findings via both endoscopic methods among a few selected subjects.

**Figure 1:**
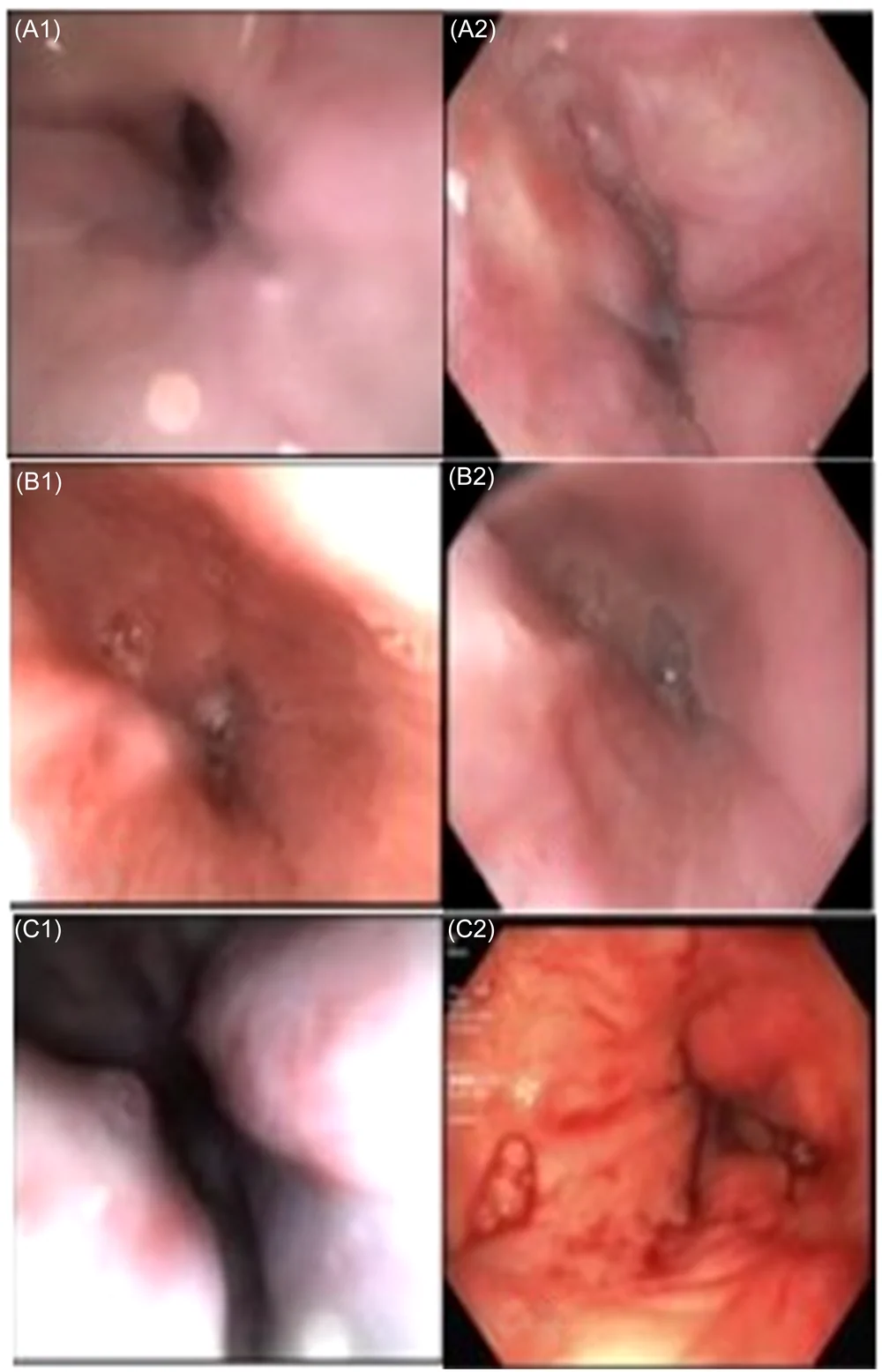
Esophageal findings via single‐use ultraslim gastroscope and standard gastroscope. Grade I varices identified via ultraslim endoscope (A1) and standard gastroscope (A2). Grade II varices identified via ultraslim endoscope (B1) and standard gastroscope (B2). Grade III varices identified via ultraslim endoscope (C1) and standard gastroscope (C2).

Three subjects with high‐risk varices required intervention with either sclerotherapy (*n* = 1) or band ligation (*n* = 2). The remaining subjects required no endoscopic intervention. However, 9/10 subjects had either sclerotherapy or band ligation previously performed, and 3/10 subjects were on non‐selective beta blockers for medical management of their EV. No patient experienced any complications or adverse events from the endoscopic procedures.

## 4 DISCUSSION

Visual imaging is of utmost importance in identifying and grading EV. Prior to initiating sedation‐free TNE for EV, a query about the adequacy of such scopes being used for evaluation and grading of EV was performed. Ten subjects underwent routine sedated surveillance endoscopy for EV with a recently FDA‐cleared 3.5 mm outer diameter single‐use endoscope and a standard larger diameter (>8.6 mm) gastroscope on the same day to evaluate visual congruence for presence and grading of EV. The study found that the ultra‐slim endoscope and the standard of care large diameter oral gastroscope had 100% visual concordance for the presence or absence of EV. Among the eight subjects found to have EV, 87.5% had matching variceal grades between both devices. These results suggest that the ultra‐slim endoscope can identify and grade pediatric EV with high concordance compared to the standard‐of‐care endoscopes.

Precise identification and classification of EV guide the management of pediatric EV. Our study demonstrates the potential for considering TNE for EV evaluation in a lower cost, lower acuity setting.^13^, ^14^ A 100% concordance in variceal grading between devices among patients undergoing endoscopy for primary prophylaxis supports the use of sedation‐free TNE for primary prophylaxis of EV. In our cohort, 60% of patients undergoing endoscopy for secondary prophylaxis did not require therapeutic intervention, highlighting the potential to expand the use of TNE for secondary prophylaxis. This highlights the importance of patient selection and that this method of screening could be indicated in patients with a distant history of variceal bleed and/or stable, low‐risk EV findings in between. TNE also offers a cost‐effective, accessible option for resource‐limited settings and for pediatric patients at risk from sedation.^1^, ^9^

Given its size, ultra‐slim endoscopy is limited in its therapeutic capabilities as its 2.0 mm working channel can fit a sclerotherapy needle but lacks compatibility with banding devices.^4^, ^16^ In this study, all patients with a history of Grade II or Grade III varices or recent variceal bleeding required intervention, and thus would not have been ideal candidates for sedation‐free TNE. Thus, in the single discordant case, the patient's history of Grade III varices and recent, recurrent symptomatic bleed would place this patient at a high likelihood of recurrence of high‐risk varices and need for intervention, making sedation‐free TNE inappropriate. Furthermore, there were likely several factors, such as user variability and proximity to recent bleeding symptoms, which likely contributed to the discrepancy in variceal grading in the case. Patients with a history of recent variceal bleed or known high‐grade varices may not be ideal candidates for sedation‐free TNE.^17^, ^18^ Additionally, non‐invasive markers such as platelet count, splenomegaly, degree of liver disease, prior history of variceal bleeding, timing of variceal bleeding, prior history of high grade EV can be used to help risk‐stratify patients.^19^ High risk stigmata of recent bleeding such as red wale signs or fibrin plugs were not assessed and documented in this study, but recording of these features should be considered for observation in future studies to evaluate their role in risk‐stratification of patients. Additional research is needed to further identify strategies and patient characteristics to identify ideal candidates when considering sedation‐free TNE in the primary and secondary prophylaxis surveillance and management of EV.

Limitations of this pilot study that may limit extrapolation to sedation‐free TNE and use of ultra‐slim endoscopes may include patient position, endoscopist skill level, and sedation. Patients in this study were positioned supine, which could result in different visualization of varices in comparison to when a traditional pediatric TNE is performed in a sitting, non‐sedated patient.^8^, ^20^ It may be hypothesized that blood flow in varices may change in variant patient positions, but adult TNE has been reported to be performed successfully in both the lateral and the sitting position as compared to pediatrics.^1^, ^16^, ^20^ Feasibility and successful use of sedation‐free TNE is also dependent on comfort and technical skill of the operator. Ultra‐slim endoscopy, whether transnasal or transoral, is still a relatively new method of endoscopy, and the endoscopist may require additional training on how to best use the endoscope to recognize EV.^4^, ^20^ Furthermore, some of the technical aspects, such as the use of insufflation to classify EV, may be different, as the air flow/insufflation on the ultra‐thin scope is on/off as compared to continuous flow/enhanced flow found in the larger diameter endoscopes. This may have also contributed to the variant grading of the Grade I versus Grade II varix noted for the discordant case in this study. Finally, the use of sedation for TNE may have allowed for enhanced visualization of the esophagus and EV as compared to an awake pediatric patient who is known to swallow, be more active, and have active peristalsis during sedation‐free TNE. Further research for evaluation of EV in an awake patient is needed.

## 5 CONCLUSION

This study demonstrates that pediatric endoscopy using single‐use ultra‐slim gastroscope accurately and reliably identifies the presence of EV and grades EV with high reliability as compared to standard of care large diameter oral gastroscopes. Use of the single‐use ultraslim gastroscope holds great potential in transforming clinical practice in the evaluation and management of EV and improving the overall accessibility of care and monitoring of EV for pediatric patients with liver disease. Additional studies are required to better understand best practices, feasibility, accuracy, and potential guidelines for consideration of the use of sedation‐free TNE for monitoring of EV.

## CONFLICT OF INTEREST STATEMENT

Paul Tran is a consultant for EvoEndo and was not compensated for this study. Endoscopic devices utilized for this study were supplied by EvoEndo, Inc (Centennial, CO). Shauna Schroeder is a consultant for EvoEndo and was not compensated for this study. Joel A. Friedlander is Chief Medical and Innovation Officer of EvoEndo, Inc. He is an employee, board member, stockholder, and is listed as co‐inventor on several patents related to endoscopic technology, methods, and virtual reality technology. The remaining authors declare no conflict of interest.
